# Hair Cortisol Measurement by an Automated Method

**DOI:** 10.1038/s41598-019-44693-3

**Published:** 2019-06-03

**Authors:** Diego Gonzalez, Dario Jacobsen, Carolina Ibar, Carlos Pavan, José Monti, Nahuel Fernandez Machulsky, Ayelen Balbi, Analy Fritzler, Juan Jamardo, Esteban M. Repetto, Gabriela Berg, Bibiana Fabre

**Affiliations:** 1 Universidad de Buenos Aires, Facultad de Farmacia y Bioquímica, Departamento de Bioquímica Clínica, Catedra de Bioquímica Clínica I, Buenos Aires, Argentina; 2 Universidad de Buenos Aires, Facultad de Farmacia y Bioquímica, Instituto de Fisiopatología y Bioquímica Clínica (INFIBIOC), Buenos Aires, Argentina; 3 Universidad de Buenos Aires, Consejo Nacional de Investigaciones Científicas y Técnicas (CONICET), Facultad de Farmacia y Bioquímica, Buenos Aires, Argentina; 4 Universidad de Buenos Aires, Consejo Nacional de Investigaciones Científicas y Técnicas (CONICET), Instituto de Química y Fisicoquímica Biológicas (IQUIFIB), LANAIS-PROEM, Buenos Aires, Argentina

**Keywords:** Steroid hormones, Adrenal cortex hormones

## Abstract

We present the development of the first procedure for hair cortisol measurement through an automated method. Hair samples were obtained from 286 individuals. After cortisol extraction, samples were measured in a Siemens Immulite 2000 (Gwynedd, UK) automated chemoluminiscent immunoassay analyzer. Normal reference values were obtained from hair cortisol levels measured in 213 healthy individuals with low levels of stress. Hair cortisol concentration median was 55 pg/mg hair (2.5–97.5 percentile (40–128)) in healthy individuals with low levels of stress and 250 pg/mg hair (range 182–520) in stressed individuals. No significant differences were observed in hair cortisol levels between subjects with and without dye (40 (40–107) and 40 (40–155) pg/mg hair, respectively; p = 0.128). The novel procedure presented here shows an adequate analytical performance.

## Introduction

The study of chronic hyperactivity of hypothalamic-pituitary-adrenal (HPA) axis in different clinical situations such as chronic stress and other pathologies related to HPA axis is difficult due to the lack of a suitable biomarker^1–4^. Given that cortisol is the recommended hormone for the evaluation of HPA axis, its highly variable circulating concentrations due to pulsatile secretion and circadian rhythm should be considered^5^. Cortisol measurement in serum, saliva and urine represents cortisol levels at one specific moment. Although saliva is considered a non-invasive, stress-free sample and applicable to different populations (pediatric, psychiatric, geriatric), several investigations show a lack of association with chronic stress and different HPA pathologies^6–8^.

Clinical laboratories show an arising necessity of finding a biological sample capable of evaluating cortisol levels increase and reflecting chronic stressors impact on human health. This is the reason why several researches propose different biological matrices that allow the estimation of systemic hormone concentration over time. There is growing evidence that attributes this advantage to hair sample. As previously described, free cortisol fraction diffuses from blood capillary to the growing hair follicle and is incorporated into hair, where it remains unaltered^9^. Since hair grows approximately 1 cm per month, it is claimed that 3 cm of hair would reflect cortisol levels to which the individual was exposed in the last 3 months^10^. Some studies have reported that hair cortisol levels may reflect HPA axis activity and correlate with different stressors^11^. Moreover, hair cortisol levels would allow to evaluate the clinical impact of Cushing’s syndrome (CS), subclinical CS, and cyclic CS in clinical endocrinology laboratory. Hair cortisol measurement could also become a useful biomarker in the follow-up of patients with adrenal insufficiency treated with hydrocortisone and could constitute a better option to evaluate HPA axis in different pathophysiological situations associated with chronic stress^12,13^.

It is important to note that so far, no automated methods have been developed for the determination of hair cortisol, being mass spectrometry (MS) the most commonly used method^14^. However, it must be considered that MS is an expensive and low accessibility method to clinical laboratories in our country. Besides, automated systems allow a faster response in a large number of samples with high analytical precision and sensitivity. It is, therefore, of great interest to incorporate an automated method for hair cortisol measurement to clinical-endocrinology laboratories. The aim of this study was to develop and evaluate a procedure to measure hair cortisol through an automated method available in any clinical-endocrinology laboratory and to compare this procedure with MS.

## Materials and Methods

### Hair collection and sample preparation

Hair samples were obtained with scissors from the posterior vertex as close to the scalp as possible. Considering that hair grows approximately 1 cm per month, 3 cm were obtained in order to evaluate hair cortisol levels representative of the last 3 months. Each sample was kept in aluminum foil at room temperature until it was processed. Fifty milligrams of hair were weighted on an analytical scale, cut into small pieces with scissors, placed in a glass tube with emery boards and 4 mL of methanol were added. The tube was sealed with a cap and incubated for 3 h in a shaker at room temperature and then overnight at 50 °C for steroid extraction. After incubation, tubes were spinned for 1 minute in a vortex and 1.2 mL of supernatant were collected in Khan glass tubes and evaporated until dryness at room temperature (48 h). The dry remnant was reconstituted by adding 300 µL of the diluent recommended by the manufacturer, vortexed for 1 minute and cortisol was measured in the autoanalyzer.

### Analytical performance

The extracted cortisol was measured in a Siemens Immulite 2000 (Gwynedd, UK) automated chemoluminiscent immunoassay (CLIA) with minor modifications. We performed a new calibration curve with dilutions of cortisol calibrator solutions in a range of 5.3–42 nmol/L (0.19–1.5 µg/dL) using CLIA’s kit diluent recommended by the manufacturer. Calibration solutions were obtained from RIA kit, Immunotech Beckman Coulter (catalog number: IM1841), which were calibrated against the reference preparation ERM-DA192 and 193. Logit-log calibration curves were constructed and sample values were interpolated into these curves to determine cortisol concentrations. In order to evaluate CLIA analytical performance for hair cortisol measurement, matrix effect was studied comparing the slopes of two calibration curves, one constructed with CLIA’s kit diluent and the other one with hair extract. Moreover, limit of blank (LOB), limit of detection (LOD), and limit of quantification (LOQ) were assessed according to Clinical and Laboratory Standards Institute (CLSI) EP17-A2 guideline^15^. LOB was established as the 95th percentile from repeated measurements (60 times) of a blank sample (CLIA’s kit diluent). LOD was established from repeated measurements of three sample pools with concentrations between LOB and four times LOB. LOQ was determined from a precision profile using four hair cortisol samples of different concentrations. Intra and inter assay coefficients of variation (CV) were evaluated in two different hair cortisol concentrations, 40 and 171 pg cortisol/mg hair. In order to evaluate within-subject variation, two different hair samples obtained from two proximal areas in the posterior vertex were analyzed. In the recovery assay, hair samples with known cortisol concentrations were supplemented with 0.38 µg/dL, 0.75 µg/dL and 1.5 µg/dL of cortisol standard. Each determination was performed in quadruplicate. Hair extract stability after adding 300 µL of diluent was tested in two different concentrations (163 pg/mg hair and 263 pg/ mg hair) every 48 hours at 4 °C for 8 days and at −20 °C for 5, 10, 20 and 30 days.

### Cross reactivity

The cross reactivity, as reported by the manufacturer, is as follows: after adding 400 µg/dL of corticosterone and cortisone, the cross reactivity was 1.2% and 1% respectively; after adding 100 µg/dL of deoxicortisol, the cross reactivity was 1.6%, and after adding 8 µg/dL of prednisolone, the cross reactivity was 62%. No cross reactivity was observed with estradiol, estrone or dexamethasone.

### Interference testing

#### Effects of hair dye on hair cortisol levels

In order to evaluate the effect of dye, two procedures were performed. In the first procedure hair cortisol levels were measured in two samples obtained at the same time from the same subject, one devoid of dye and another one previously dyed once a month for the last three months. In the second procedure hair cortisol levels were measured in two groups of individuals, with and without hair dyeing (n = 126 and n = 106, respectively).

### Effect of washing and other interferences in hair cortisol measurement

Five hair samples were selected at random and divided in two portions. One portion of each sample was processed with the proposed method and the other portion was previously washed with isopropanol. This latter samples were washed with 2 mL of isopropanol in glass tubes under slowly rotation for 2 minutes. Then, isopropanol was decanted and hair was dried overnight at room temperature.

Besides, in order to investigate possible interferences due to the use of dandruff shampoo 500 µL of shampoo were processed under the same conditions than hair, and cortisol was measured with the proposed method. In parallel, 3 cm of hair were washed *in vitro* with shampoo, carefully rinsed with tap water, dried, extracted and finally cortisol was measured.

### Comparison with MS

The automated method was compared with MS in 10 hair samples.

Prior to the analysis, each dried sample obtained according to the sample preparation was reconstituted in 20 µL of solvent A (2% acetonitrile and 2% acetic acid) during 5 minutes of treatment in a bath sonicator. The final concentration was 60 µg/dL (1.6 µM).

The liquid chromatography (LC) system consisted of a Surveyor MS pump online with the mass spectrometer. A reverse phase C4 30 × 1 mm column from Higgins Analytical (Proto 300 C4 5 um RS-0301-W045), and solvents A and B (96% acetonitrile and 2% acetic acid) for the mobile phase was used. The gradient applied after equilibrium for at least 10 min in 2% B consisted in three isocratic steps: 2% B from 0 to 5 min, 100% B from 5.1 to 13 min and 2% B from 13 to 21 min at a flow rate of 40 µL/min. All solvents and reagents were HPLC or LC grade.

An LCQ Duo (ThermoFisher) was used for all data collection. Electrospray voltage of 4.5 kV was applied at the source with a sheath gas flow rate of 45 arbitrary units. The ion transfer tube was set to 200 °C. A full MS scan range of 150–2000 was used for all samples. The Automated Gain Control (AGC) target and maximum injection time were set at 5 • 10^7^ counts and 200 ms, respectively. Data-dependent acquisition mode was used to trigger cortisol precursor isolation and characterization. The collision energy was set to 35%, and the threshold was set to 8 • 10^4^ counts. The mass width was set at 1. The MS full scans and the MS/MS scans were recorded by a method that allows the acquisition of an MS/MS spectra after each MS full scan.

All raw files were analyzed with the Xcalibur 1.3 software. For positive cortisol identification, we compared retention time and MS/MS spectra with the one obtained with cortisol standard solution.

### Study population for reference values

In order to set up reference interval we included 232 healthy individuals (30 men (41 ± 12 years) and 202 women (45 ± 15 years)) without medication or adrenal pathology. These were divided into two groups: individuals with high (stressed individuals) and with low levels of stress, according to Holmes- Rahe scale score^16^. Holmes-Rahe schedule of readjustment scale was self-administered and included 40 items where individuals indicated which event they experienced during the past 1–5 years.

The normal reference interval was obtained using percentile (P) 2.5 and 97.5 from hair cortisol levels measured in 213 healthy individuals who presented a Holmes-Rahe life events scale score <300^17,18^. The remaining 19 individuals with Holmes-Rahe life events scale score >300 were considered stressed individuals.

The participants in this study did not receive any kind of compensation for participating and all of them gave written prior informed consent. The study was approved in advance by Hospital Ethic Committee (Comité de Etica del Hospital de Clínicas José de San Martín, Universidad de Buenos Aires) and was performed following the Helsinki Declaration for medical studies in humans.

### Statistical methods

Results are expressed as mean ± standard deviation (SD) or median (range) according to data distribution (Shapiro-Wilk test). Mean and median differences were evaluated by t-test or Mann-Whitney test, respectively. Repeated measures analysis of variance (ANOVA) was performed to study hair extract stability over time. Chi-Square test was performed to compare hair cortisol levels in different clinical situations. A p-value of less than 0.05 was considered as statistically significant. The Statistical Package for Social Sciences (SPSS: version 23.0) was used for data analysis.

## Results

### Automated chemiluminescent assay analytical performance for hair cortisol

**Figure 1:**
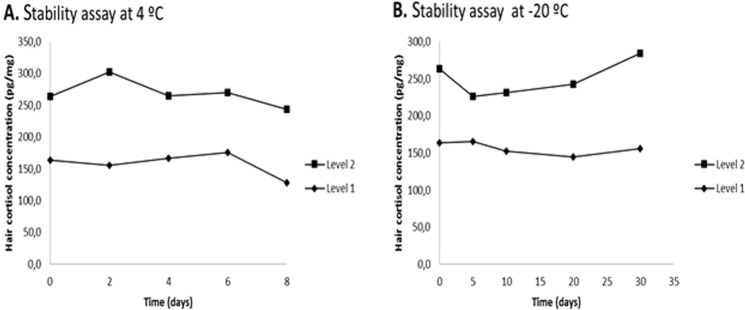
Stability assay of the reconstituted hair extract at 4 °C and −20 °C. (**A)** Stability in two concentrations (Level 1: 163 and Level 2: 263 pg/mg hair) at 4 °C (F = 1.72, p = 0.306 and F = 1.22, p = 0.436, respectively). (**B**) Stability in two concentrations (163 and 263 pg/mg hair) at −20 °C (F = 0.60, p = 0.68 and F = 2.34, p = 0.21, respectively).

Calibration curve slopes corresponding to the matrix effect assay were −1.978 (95% CI, −2.231 to −1.726; y = 0.8472–1.978x; r^2^ = −0.953) with CLIA’s diluent and −2.099 (95% CI, −2.324 to –1.874; y = 0.7353–2.09977x; r^2^ = −0.9662) with hair extract, finding non-significant differences. The obtained LOB was 0.9 nmol/L (0.03 µg/dL), LOD was 2.0 nmol/L (0.07 µg/dL) and LOQ was 5.6 nmol/L (0.20 µg/dL) of cortisol which corresponds to 40 pg cortisol/mg of hair. Intra and inter assay CV were 15% and 20% for 40 pg cortisol/mg of hair respectively and 11% and 14% for 171 pg cortisol/mg of hair. Within-subject CV obtained from two different hair samples was 17%. Cortisol mean analytical recovery was 81.6%, 87.5%, and 99.5% for 0.38 µg/dL, 0.75 µg/dL and 1.5 µg/dL of cortisol standard, respectively. Stability test showed that reconstituted hair extract was stable up to 8 days at 4 °C and 30 days at −20 °C (Fig. 1).

### Interference testing

#### Effects of hair dye on hair cortisol levels

Regarding the effect of hair dyeing, no differences were observed in cortisol levels in samples obtained from the same individual comparing dyed (86 pg/mg) and undyed (98 pg/mg) hair. Furthermore, no significant differences were observed in hair cortisol levels between subjects with and without hair dye (40 (40–107) and 40 (40–155) pg/mg, respectively; p = 0.128).

#### Effect of washing and other interferences in hair cortisol measurement

Similarly, no differences were found when comparing hair samples with and without previous *in vitro* washing with isopropanol (119 (86–588) and 106 (58–500) pg/mg, respectively; p = 0.38). The addition of dandruff treatment shampoo to hair samples revealed no differences in hair cortisol levels with and without treatment (101 *vs* 95 pg/mg). Finally, cortisol concentration measured in a dandruff shampoo aliquot extracted with the same procedure as hair samples was 206.9 nmol/L (7.5 μg/dL).

### Comparison with MS

**Figure 2:**
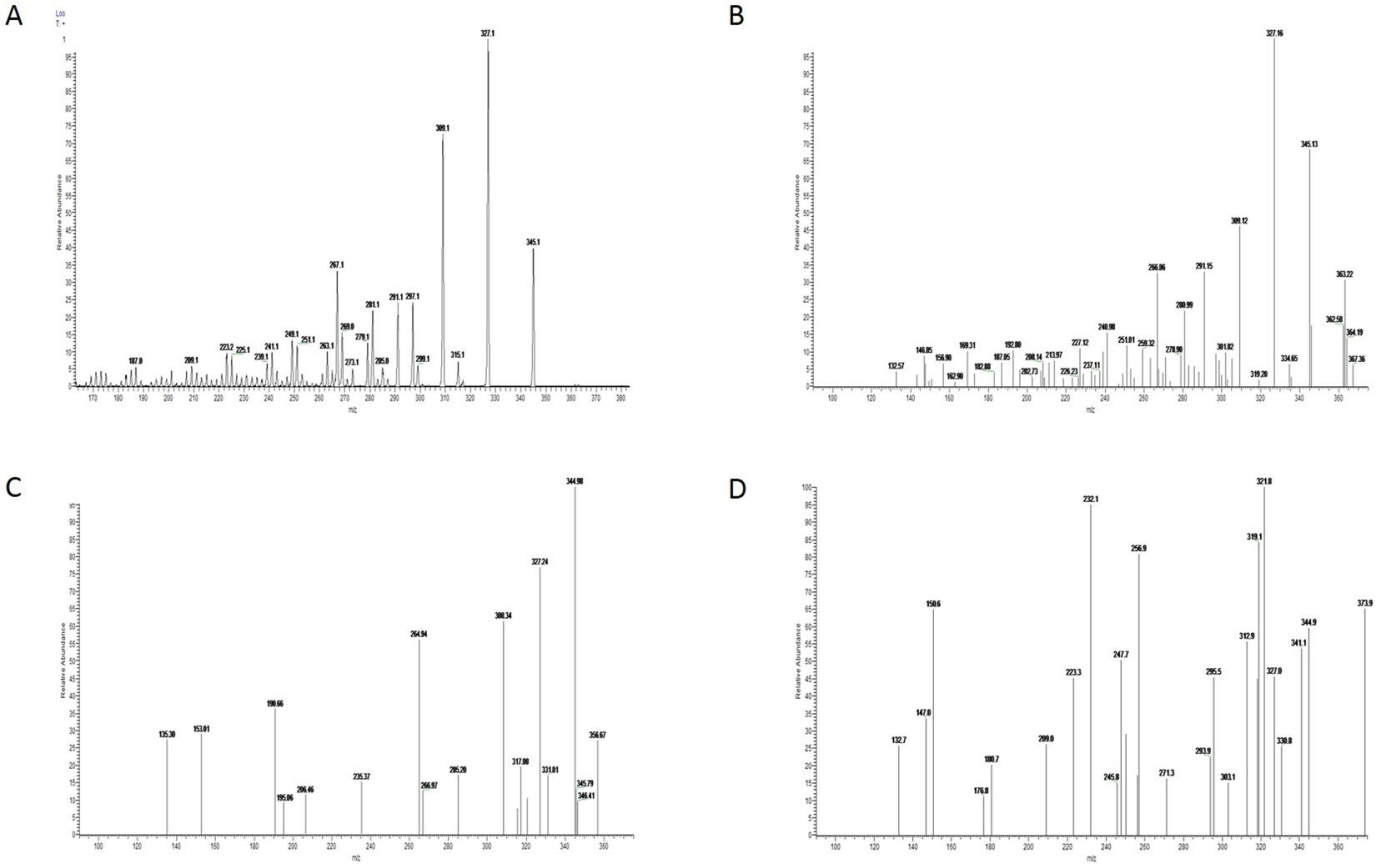
MS/MS spectra for the (M + 1H)^+1^ ion at m/z 363. (**A)** Cortisol reference material; (**B**) sample matching cortisol characteristic profile; (**C**) sample with incomplete match to the cortisol profile and therefore considered “Negative”. (**D)** Sample that clearly does not match the cortisol profile.

Cortisol presence in methanol extracts was verified by tandem MS. Cortisol characteristic signal was observed in six samples in which it was also possible to quantify cortisol by the automated method with concentrations ranging between 40 and 108 pg/mg. Moreover, in four samples in which cortisol signal was not observed by MS, the automated method reported concentrations lower than the LOQ (Fig. 2).

### Hair cortisol reference interval

The characteristics of the healthy study population (n = 232) are shown in Table 1. No differences in hair cortisol were observed considering age, gender, education, occupational or marital status, use of contraceptive drugs or physical activity.

**Table 1:** Sociodemographic and general characteristics of the studied population.

Variables		n (%)
Gender	Women	202 (87)
	Man	30 (13)
Age (years)	18–24	12 (5)
	25–40	95 (41)
	41–85	125 (54)
Education level	Primary school	5 (2)
	Secondary school	60 (26)
	Tertiary level	39 (17)
	University degree	128 (55)
Employment status	Unemployed	7 (3)
	Employed	190 (82)
	University student	35 (15)
Marital status	Married	83 (36)
	Divorced	6 (3)
	In a relationship	74 (32)
	Unmarried	67 (28)
	Widower	2 (1)
Contraceptive therapy	No	192 (83)
	Yes	40 (17)
Physical activity	No	144 (62)
	Yes	88 (38)

**Figure 3:**
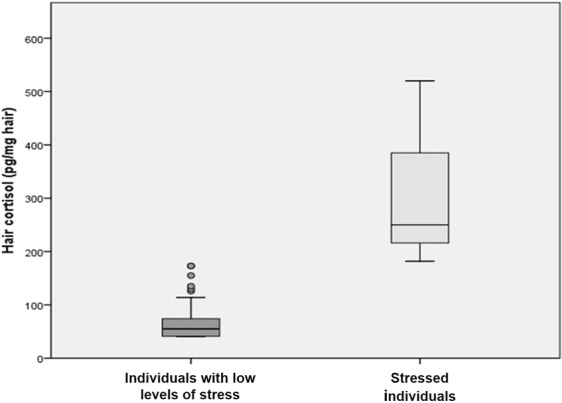
Hair cortisol concentration in stressed and individuals with low levels of stress. Hair cortisol levels in healthy individuals with low levels of stress (40–128 pg/mg; n = 213) and in stressed individuals (182–520 pg/mg; n = 19); p < 0.05.

The obtained hair cortisol concentration reference interval in healthy individuals with low levels of stress was 40–128 pg/mg hair (P2.5-P97.5). Hair cortisol concentrations in stressed individuals were higher than in the group with low levels of stress 250 (182–520) pg/mg hair (p < 0.05) (Fig. 3).

## Discussion

In this study, we describe the first automated procedure for the measurement of cortisol levels in human hair. The novel technic presented in this work represents an original, economic, available method, allowing the assessment of several samples at the same time.

Nowadays, hair cortisol is considered an appropriate biological marker for chronic stress^10,19^ and also a biomarker of alterations in HPA axis such as cyclic CS and adrenal insufficiency^2,19–22^. The most commonly used method for hair cortisol evaluation is MS although its application in clinical laboratories in our country is limited. Alternatively, a salivary Enzyme-Linked Immunosorbent Assay (ELISA) non-automated method has been proposed for hair cortisol measurement. Still, this alternative method allows the evaluation of a limited number of samples and presents high coefficients of variation which are typical in manual procedures.

With the procedure described in this study, we evaluated hair cortisol levels in individuals with dyed and undyed hair and no statistical differences were found, either in dyed or undyed hair belonging to the same individual. Previous studies found decreased hair cortisol levels measured with an ELISA method when hair was *in vitro* dyed^10,14^, but other studies applying MS found non-significant differences^23^, in agreement with our results.

Regarding the effect of washing hair samples previously with isopropanol, no differences in hair cortisol levels were found in our study. This result is in accordance with previous researches^23^, however, others have described a decrease in hair cortisol levels, when hair was washed excessively^24^. High cortisol levels were found in dandruff shampoo confirming the presence of an interference. Even though, *in vitro* hair treatment with this shampoo did not increase hair cortisol levels. Strikingly, hair cortisol levels of current users of this shampoo revealed high hair cortisol levels, probably as a consequence of its routine use.

The procedure presented in this study was compared with MS, verifying the presence of cortisol in the samples after the extractive procedure described for automated analysis. Moreover, those hair samples, measured by the proposed automated method, with cortisol concentrations below LOQ were also not detected by MS.

The reference interval for the novel procedure was obtained from healthy individuals with low levels of stress, in accordance with those reported by Sauve *et al*.^10^ and Raul *et al*.^25^ using ELISA and MS, respectively.

Hair length could represent a limitation for hair cortisol measurement with the automated method, especially in men, who mostly present short hair or are bald. In these cases, an alternative matrix should be used.

The novel procedure presented here shows an adequate analytical performance, providing the advantage of allowing the measurement of a high number of samples in a short time and with low cost.
